# Annotations

**Published:** 1907-11-30

**Authors:** 


### ANNOTATIONS

### The Diagnosis of Rheumatic Fever.

THE terms "rheumatism" and "rheumatic fever" have suffered much criticism in the course of medical history, and it cannot be said, even yet, that they have reached a secure position. While a large number of affections of the joints have been removed from their province, many conditions, more especially as occurring in childhood, are now regarded as evidences of rheumatism which formerly were unsuspected of any such association. There has thus been movement in the direction both of restriction and extension. In an address recently delivered to the British Balneological Society, Dr. Norman Moore proposes to push the policy of restriction in a somewhat vigorous fashion. He contends that rheumatic fever is a disease dependent on a micro-organism, a view which doubtless commands widespread sympathy. But, in addition, he suggests that the central place of growth of this micro-organism is the endocardium, and that it is from this central source that the joints and other tissues become infected. The logical outcome of such a position obviously is that without endocarditis there is no rheumatic fever, and Dr. Moore does not hesitate to apply this argument to both diagnosis and treatment. Cases of joint pains with raised temperature, he says, in which, after daily observation for a period of three weeks, no sign of endocarditis can be discovered, should be excluded from the description rheumatic fever. This is certainly a very radical proposal, and one which will hardly command ready adoption. Statistics, it is true, differ very much regarding the frequency with which endocarditis occurs in acute rheumatism, but no observer, so far as we know, has claimed more than that a considerable proportion are so affected. If the new proposal is adopted, what is to become of the minority who escape? Of course, it may be said that such cases must be separated from acute rheumatism just as other acute affections of the joints have been separated. That may be possible as a matter of doctrine, but in the absence of any alternative ætiology for such cases it is hardly possible in practice. To emphasise the extreme liability to endocarditis in all manifestations of rheumatism is in the highest degree desirable, but we question whether the present position of clinical knowledge will justify the aphorism "No endocarditis, no rheumatism."

### London's Water Supply.

AT a meeting of the Metropolitan Water Board held on November 22 an interesting statement was submitted on behalf of a committee charged with the consideration of the future water-supply of London. Mr. E. B. Barnard, the chairman of the committee, said that it was necessary to consider the provision of an adequate water-supply in view of what might be anticipated during the next fifty years. Accepting the estimate of the Royal Commissions of 1892 and 1897 in regard to the consumption per head per day as 35 gallons, and adopting the calculation that in 1961 it would be necessary to provide for sixteen millions of people, 550 millions of gallons of water per day would then be needed. This means an addition of 265.5 millions of gallons to the present daily supply. To meet this demand the committee recommends, in the first place, that powers should be obtained to procure additional supplies from the Thames, going for this purpose higher up the river, and thus obtaining a better and safer quality of water. Looking still further ahead, the Comittee emphasises the necessity of acquiring an auxiliary gathering ground for use after increased supplies from the Thames are no longer economically available. Speaking of the quality of the existing water-supply, Mr. Barnard claimed that this has undergone a continuous improvement, largely owing to more efficient filtration, and he believed that even still better results were to be expected. On the question between hard and soft waters he stated that in towns supplied with the former the death-rate was 16.5, whilst in those provided with soft water it was 19.2. In the same spirit he contended that in regard to infectious diseases towns which depended on hard water were more healthy than places which had a soft-water supply. These figures are, of course, interesting, but the particular issues to which they are here applied are somewhat too complex to be settled by statistics which deal only with a few of the facts.

### Aberdeen University Club, London.

THE usual semi-annual dinner of this club was given in the New Gaiety Restaurant, Strand, on Wednesday, November 20, when Mr. W. Watson Cheyne, C.B., F.R.S., took the chair. There was a large and representative gathering of the members of the club and of their guests, and the meeting was in every sense a highly successful one. After the usual loyal toasts had been duly honoured, the Chairman proposed "The University of Aberdeen and the Aberdeen University Club." In so doing he referred to the recent extension of the provision for laboratory teaching and research which had taken place at Marischall College, and expressed the conviction that the University had before it a sure prospect of continued success and usefulness. Referring to his own early association with Aberdeen, he quoted a number of interesting reminiscences of former professors, and alluded to the success which had marked the careers of a number of his contemporaries. He also paid a warm tribute to the work of the surgical school in Aberdeen, and particularly to the influence and teaching of Professor Ogston. The London club he regarded as a valuable organisation, inasmuch as it kept alive traditions of which Aberdeen men had reason to be proud, and gave an opportunity for the cultivation of mutual fellowship and goodwill. The toast of "The Guests" was proposed by Dr. C. O. Hawthorne, and, in replying, Sir Thos. Barlow expressed his admiration of the solidarity which marked the work and tone of the northern University, and regretted that a similar spirit had not yet been aroused in London. Dr. Geo. Ogilvie proposed, in his usual successful manner, the health of "The Chairman," and Mr. Watson Cheyne made an appropriate reply. Amongst the musical features of the evening were some much-appreciated Scottish ballads given with great spirit by Mr. Jas. Cantlie.

